# Controversies and insights into PTBP1-related astrocyte-neuron transdifferentiation: neuronal regeneration strategies for Parkinson’s and Alzheimer’s disease

**DOI:** 10.1186/s40035-024-00450-9

**Published:** 2024-12-03

**Authors:** Simon McDowall, Vaishali Bagda, Stuart Hodgetts, Frank Mastaglia, Dunhui Li

**Affiliations:** 1 Perron Institute for Neurological and Translational Science, Nedlands, WA Australia; 2 School of Human Sciences, The University of Western Australia, Crawley, Perth, WA Australia; 3 Department of Anatomy and Department of Pathology, University of California San Francisco, San Francisco, CA USA; 4 Centre for Molecular Medicine and Innovative Therapeutics, Health Futures Institute, Murdoch University, Murdoch, WA Australia; 5 Centre for Neuromuscular and Neurological Disorders, Nedlands, WA Australia; 6 Department of Neurology and Stephen and Denise Adams Center for Parkinson’s Disease Research, Yale School of Medicine, New Haven, CT USA

**Keywords:** Neuronal regeneration, Astrocyte-neuron reprogramming, Astrocyte heterogeneity, Therapeutic strategies

## Abstract

Promising therapeutic strategies are being explored to replace or regenerate the neuronal populations that are lost in patients with neurodegenerative disorders. Several research groups have attempted direct reprogramming of astrocytes into neurons by manipulating the expression of polypyrimidine tract-binding protein 1 (PTBP1) and claimed putative converted neurons to be functional, which led to improved disease outcomes in animal models of several neurodegenerative disorders. However, a few other studies reported data that contradict these claims, raising doubt about whether PTBP1 suppression truly reprograms astrocytes into neurons and the therapeutic potential of this approach. This review discusses recent advances in regenerative therapeutics including stem cell transplantations for central nervous system disorders, with a particular focus on Parkinson’s and Alzheimer’s diseases. We also provide a perspective on this controversy by considering that astrocyte heterogeneity may be the key to understanding the discrepancy in published studies, and that certain subpopulations of these glial cells may be more readily converted into neurons.

## Introduction

Neurodegenerative diseases are the leading cause of cognitive and physical disability worldwide, affecting over 15% of the population [1] . As life expectancy increases, the incidence of conditions such as Alzheimer’s disease (AD) and Parkinson’s disease (PD) is predicted to double in the coming decades, posing a significant challenge to the healthcare system [2–4]. Loss of dopaminergic neurons in the substantia nigra pars compacta is the histopathological hallmark of PD, which contributes to the cardinal motor symptoms including bradykinesia, rigidity, tremor, and postural instability [5, 6]. The rapid progression of PD often results in exacerbated symptoms that significantly compromise the quality of life, cause cognitive impairments, and increase the mortality rate in affected individuals [7, 8].

AD is recognized as the most prevalent neurodegenerative disorder, affecting around one in 10 people over the age of 65, and accounting for ~ 70% of all cases of dementia [9, 10]. AD is characterized by aggregation of amyloid beta and phosphorylated tau proteins in the hippocampus and other brain regions responsible for memory, spatial navigation, language, and higher-order cognitive functioning [4, 11]. In addition, intracellular neurofibrillary tangles arise within neurons as a result of abnormal phosphorylation of tau protein, leading to the degeneration of pyramidal neurons in affected cortical regions [12]. Consequently, patients with AD commonly experience significant cognitive impairments such as memory loss, difficulties with executive functioning, and lack of spatial awareness, which can result in increased mortality rates at advanced stages of the disease [13].

In 1928, Ramón y Cajal stated that “in adult centers, the nerve paths are something fixed, ended, and immutable. Everything may die, nothing may be regenerated. It is for the science of the future to change” [14]. This doctrine for neuroscience became a call-to-action of sorts for the development of innovative approaches to replace or regenerate damaged or degenerated neurons in the brain.

Despite advances in technology and in our understanding of the mechanisms underlying such diseases, no therapies have been proven to modify the disease course and current available treatments can only provide temporary symptom relief for patients. Several neuro-regenerative and disease-modifying approaches are being explored, including stem cell therapy and cellular reprogramming using transcription factors. However, these approaches have been met with concerns including tumorigenicity, difficulty with resourcing of cells, high costs, and prolonged technical procedures [15–17], impeding the translation of these therapeutic strategies into the clinic.

Emerging studies have demonstrated the feasibility of direct conversion of glial cells to neurons by regulating levels of factors including polypyrimidine tract-binding protein 1 (PTBP1), NEUROD1, and SOX2, resulting in significant functional recoveries in disease models [18–27]. However, conflicting results were reported in several recent studies, particularly regarding downregulating PTBP1 to reprogram glia into neurons in vivo [28–30]. In this article, we will provide a comprehensive review of current neuro-regenerative strategies for PD and AD, with a particular focus on glial-neuronal transformation and the debate surrounding PTBP1*-*mediated reprogramming of glial cells. We will also discuss how the complexity of astrocyte populations likely underlies these discrepancies in the existing literature [31, 32], and provide our insights into the development of novel and effective neuro-regenerative approaches using available therapeutic modalities.

## Current status of stem cell regenerative therapies

Stem cell therapies represent a promising frontier in the field of regenerative medicine and are currently considered a leading approach in the development of therapies for the repair of the CNS. Pluripotent stem cells are particularly attractive due to their ability to proliferate and differentiate into cells of all three germ layers. Stem cells are broadly categorized into two main types: embryonic stem cells (ESCs) and induced pluripotent stem cells (iPSCs) from either autologous or allogenic cell sources [32]. However, ESCs are facing many concerns surrounding the ethical sourcing of these cells and safety issues caused by immune response of allogenic transplantations [33, 34].

Autologous transplantations of a patient’s fibroblast-derived iPSCs that have identical features and developmental progression as ESCs [35], may have advantages by overcoming the immune system response [36]. However, autologous iPSCs may still retain the mutations or risk factors for PD or AD [37]. The generation of a personalized, clinical-grade cell line is extremely expensive, costing approximately $800,000 US dollars [38–40]. In addition, tumorigenesis following iPSC transplantations is another significant safety concern which requires careful and sustained post-transplantation monitoring [41].

Alternative strategies have shifted to mesenchymal stem cells (MSCs), which are adult stem cells commonly derived from sources such as bone marrow or adipose tissue [42, 43]. Through cell-to-cell contact and by secreting soluble factors including cytokines and growth factors, MSCs can modulate the proliferation and function of immune cells such as T cells, B cells, and dendritic cells [43], resulting in suppression of excessive inflammation and promotion of tissue repair, thereby reducing the risk of rejection, even in an allogenic host [44]. However, concerns regarding the long-term survivability of transplantations [45] and non-zero risk of tumorigenesis in vivo [46, 47] still exist. Combined strategies have been explored to increase the graft survivability, such as repeated growth factor injections, rehabilitation, and scar-degrading enzymes to increase axon regeneration [48]. Furthermore, efforts have been made to reduce tumor formation and to increase the safety and efficacy of stem cell transplantations, which have been recently reviewed elsewhere [49]. Some promising results have been shown in clinical trials using stem-cell based therapies for both AD and PD [50], and a number of trials are currently in progress for both diseases as shown in Table 1.

**Table 1:** Current clinical trials using stem cell-based therapies for Alzheimer’s disease (AD) and Parkinson’s disease (PD)

NCT Number	Study design	Stem cell type	Status	Estimated completion date	Disease
NCT05667649	Phase I	Autologous adipose derived MSC	Recruiting	March, 2025	AD
NCT04684602	Phase I/II	Allogenic amniotic and umbilical cord tissue-derived MSC	Recruiting	December 09, 2030	AD
NCT04482413	Phase IIb	Autologous adipose tissue-derived MSC	Not yet recruiting	December 20, 2024	AD
NCT03899298	Phase I	Allogenic amniotic and umbilical cord tissue-derived MSC	Not yet recruiting	March 20, 2029	AD
NCT03724136	N/A	Autologous BMSC	Enrolling by invitation	October, 2025	AD
NCT02899091	Phase I/IIa	Allogenic placenta-derived MSC	Active, not recruiting	December, 2024	AD
NCT02833792	Phase IIa	Allogenic human MSC	Recruiting	December 31, 2024	AD
NCT02795052	N/A	Autologous BMSC	Recruiting	July 31, 2026	AD
NCT06482268	Phase I/II	Allogenic iPSC-derived dopaminergic progenitor cells	Recruiting	May, 2028	PD
NCT06344026	Phase I/IIa	Autologous iPSC-derived dopaminergic progenitor cells	Enrolling by invitation	April 30, 2030	PD
NCT06167681	Phase I/II	Allogenic iPSC-derived dopaminergic progenitor cells	Recruiting	July, 2029	PD
NCT06145711	N/A	Autologous iPSC-derived dopaminergic progenitor cells	Not yet recruiting	December 22, 2025	PD
NCT06141317	Phase I/IIa	Allogenic adipose-derived MSC	Active, not recruiting	November 01, 2024	PD
NCT05901818	Phase I	Autologous iPSC-derived dopaminergic progenitor cells	Recruiting	December 31, 2026	PD
NCT05887466	Phase I/II	Allogenic ESC-derived dopamine progenitor cells	Active, not recruiting	February 07, 2026	PD
NCT05691114	Phase I	Allogenic human amniotic epithelial MSC	Recruiting	February, 2026	PD
NCT05635409	Phase I	Allogenic ESC-derived dopaminergic neurons	Recruiting	June, 2027	PD
NCT05152394	Phase I	Allogenic umbilical cord-derived MSC	Not yet recruiting	January, 2026	PD
NCT05094011	Phase I	Autologous adipose derived MSC	Not yet recruiting	July 31, 2026	PD
NCT04995081	Phase II	Allogenic adipose-derived MSC	Recruiting	January 15, 2026	PD
NCT02795052	N/A	Autologous BMSC	Recruiting	July 31, 2026	PD

Information retrieved from https://clinicaltrials.gov/ by the date of 3rd July 2024. Disease condition search terms on https://clinicaltrials.gov included “Alzheimer’s disease”, “Alzheimer disease”, “Alzheimer”, “Parkinson disease”, “Parkinson’s disease”, “Parkinson”. Intervention/treatment search terms included “Stem cells” and “Stem cell”. Studies that were of ‘Unknown’ status or ‘Completed” status according to clinicaltrials.gov were not included in the table. BMSC, bone marrow-derived stem cells; ESC, embryonic stem cell; iPSC, induced pluripotent stem cell; MSC, mesenchymal stem cell

## Direct conversion of cells into induced neurons using transcription factors

Direct conversion strategies may reprogram somatic cells into induced neurons without passing through a pluripotent stage, thereby avoiding concerns of tumorigenesis and are now being explored as an alternative to stem cell transplantation therapies [51, 52]. This transdifferentiation can be achieved through forced expression or repression of lineage-specific transcription factors, although it can also be achieved through miRNAs or small molecules [51, 53]. First demonstrated by Heins et al. (2002), radial glial cells isolated from mice carrying mutant *Pax6* have a significantly reduced neurogenic potential [54]. This led researchers to test forced *Pax6* expression in *Pax6-*negative astrocytes, which directed astrocytes toward neurogenesis in the embryonic and postnatal cerebral cortex [55], resulting in over half of the astrocytes differentiating into neuronal-like cells, as verified by several mature neuronal markers [55].

By harnessing these transcription factors to convert somatic cells into functional neurons, it may be possible to partially regenerate the neuronal populations that are lost in PD and AD as a therapeutic strategy to slow down or stop disease progression. This begs the question of what combination of transcription factors to be used for effective in vivo neuron reprogramming from a certain cell type*.* The combination of Brn2, Ascl1, and Myt1l was found to be required for an optimal transdifferentiation of mouse embryonic and postnatal fibroblasts to induced neurons in vitro by Vierbuchen et al. (2010) [56]. Further investigations found that non-neural lineage cells could be converted to induced glutamatergic neurons or dopaminergic neurons both in vitro and in vivo, by manipulating expression of a single factor, such as NEUROD1 [57] or PTBP1 [58]. This strategy circumvents the need for exogenous expression of transcription factors, and avoids the concerns of stem cell transplantation, offering a potential single-step conversion of glia to neurons. Recent studies have applied this strategy to astrocytes, which are an ideal cell source for in vivo reprogramming, as they are the most abundant cell type in the CNS and are proximal in developmental lineage to neurons [25, 57].

### PTBP1 suppression-mediated direct conversion of glial cells into neurons

PTBP1 was originally identified as a regulator of alternative splicing with other important roles including modulating mRNA metabolism, protein translation, and cell proliferation [59, 60]. However, it has been reported as a potential “gatekeeper” of neuronal cell identity, as changes in its expression level can result in the differentiation of peripheral cell types into functional neurons [58]. First demonstrated in 2013, downregulating PTBP1 expression by short hairpin RNAs (shRNA) induced direct conversion of multiple cell types, including HeLa cells, mouse neural progenitor cells, human retinal epithelial cells, and primary mouse embryonic fibroblasts, into functional neurons [58]. Further investigations revealed that PTBP1 inhibits a large array of neuron-specific genes including *ASCl1*, *MYT11*, *NEUROD1*, and *BRN2*, which are essential for the induction of neurogenesis in human fibroblasts [58, 61]. Thus, PTBP1 downregulation allows for these neuronal transcription factors to be activated in nonneuronal cells, thereby eliciting cellular reprogramming to functional neurons [17, 58]. Additionally, PTBP1 suppression leads to transient upregulation of PTBP2, which is seen in natural neurogenesis and is required for neuronal maturation [61]. Recent studies have reported that PTBP1 knockdown by clustered regularly interspaced short palindromic repeats (CRISPR)/CasRx, shRNA, or RNase-H1-inducing antisense oligonucleotide (ASO) is able to convert astrocytes to functional neurons, which provide axons to reconstruct the nigrostriatal circuit, restore the dopamine levels, and alleviate motor symptoms in PD mouse models in a direct, single-step process [25, 27].

## Comparison of studies reporting the efficacy of PTBP1 knockdown in converting astrocytes into neurons

As summarized in Table 2, several recent studies have claimed that glial cells can be reprogrammed into new neurons in different CNS regions via PTBP1 knockdown. In the first in vivo model by Qian et al. (2020), decreasing the level of PTBP1 using either ASOs or shRNA resulted in the restoration of striatal neurons which subsequently increased striatal dopamine, resulting in improved motor symptoms in the 6-hydroxydopamine (6-OHDA) PD mouse model [25]. However, the possibility of contributions of altered microenvironment with reduced neuroinflammation or a reduction of neurotoxic glial cells to the therapeutic benefits following PTBP1 suppression could not be ruled out. Not surprisingly, an independent study using CRISPR/CasRx to suppress PTBP1 reported a similar time-course-dependent generation of dopaminergic neurons and demonstrated similar alleviation of motor deficits in the 6-OHDA PD mouse model [27]. Additionally, a few more studies (Table 2) have reported conversion of glial cells into functional neurons in striatum, hippocampus, spinal cord, and other brain regions with functional recovery across a range of disease models following PTBP1 downregulation.

**Table 2:** Studies reporting therapeutic potential of PTBP1 knockdown

Research group & reference	Intended target cell population	Intended CNS region	Animal model(s)	Targeting vector(s)	Lineage tracing	Detection method	Target sequence(s)	Improvements in animal model?
X.D-Fu (Qian et al. (2020)) [25]	Astrocytes	Striatum	PD (6-OHDA) mice	AAV-CMV-LSL-RFP-shPtbp1	*mGfap-Cre*	Cre-dependent viral reporter	5ʹ-GGGTGAAGATCCTGTTCAATA-3ʹ (exon 10 of mouse *Ptbp1*)	Yes
		Substantia nigra pars compacta	PD (6-OHDA) mice	Anti-*Ptbp1* ASO	*hGFAP-CreER*^*T2*^*;R26R-tdTomato*	Lineage reporter	5ʹ-GGGTGAAGATCCTGTTCAATA-3ʹ(exon 10 of mouse *Ptbp1*)	Yes
H.Yang (Zhou et al. (2020)) [27]	Astrocytes	Striatum	PD (6-OHDA)	AAV-GFAP-CasRX-Ptbp1	Not done	Viral reporter	All guide RNAs can be found in Methods section	Yes
	Müller glia	Retina	NMDA injured mice	AAV-GFAP-CasRX-Ptbp1	Not done	Viral Cre-induced reporter	All guide RNAs can be found in Methods section	
D.W. Cleveland (Maimon et al. (2021)) [24]	Radial glial cells	Hippocampus	Aged WT mice	Anti-*Ptbp1* ASO1	*hGFAP-CreER*^*T2*^*;R26R-tdTomato*	Lineage reporter	5ʹ-TGCGACATTTCTCTGCACTC-3ʹ(3ʹ UTR of mouse *Ptbp1*)	Yes
				Anti-*Ptbp1* ASO2	*hGFAP-CreER*^*T2*^*;R26R-tdTomato*	Lineage reporter	5ʹ-GTGGAAATATTGCTAGGCAC-3ʹ (5ʹ UTR of mouse *Ptbp1*)	
G.Chen (Yang et al. (2023)) [26]	Spinal astrocytes	Spinal cord	Mouse model of compression-induced SCI	AAV-shPTB: AAV-GFAP-EGFP-shRNA(Ptbp1)-MIR155	Not done	Viral reporter	Same seqeuence as Qian et al. (2020)	Yes
				Anti-*Ptbp1* ASO (same sequence as Qian et al. (2020))	Not done	Neuronal density	Same seqeuence as Qian et al. (2020)	Yes
E.Huan (Yuan et al. (2024)) [78]	Astrocytes	Cerebral cortex	Mouse model of ischemic stroke	AAV-PHP.eB-GFAP-miR30-shptbp1	Not done	Viral reporter	Same seqeuence as Qian et al. (2020)	Yes
D.W.Cleveland (Maimon et al. (2024) preprint) [77]	Radial glial cells	Subventricular zone and hippocampus	Young and aged WT mice	Anti-*Ptbp1* ASO2 (same one from Maimon et al. (2021))	EdU labelling	MERFISH spatial transcriptomics	5ʹ-GTGGAAATATTGCTAGGCAC-3ʹ (5ʹ UTR of mouse *Ptbp1*)	Yes

Table adapted from Wang & Zhang (2023) [62]. 6-OHDA, 6-hydroxydopamine; AAV, adeno-associated virus; ASO, antisense oligonucleotide; MERFISH, Multiplexed error-robust fluorescence in situ hybridization; NMDA, N-methyl-d-aspartate; WT, wild type

The conversion of reactive spinal astrocytes into motor neuron-like cells in the spinal cord using a PTBP1-targeting ASO or shRNA has been recently explored for the treatment of spinal cord injury. These cells displayed a change of neuronal morphology by 2–4 weeks after PTBP1 suppression, and by 11 weeks, 19% of the newly formed neurons expressed choline acetyltransferase, a motor neuron-specific marker [26]. Motor functions were improved across a range of behavioral tests. In contrast, there was no improvement in sensory perception compared to the controls. The authors speculated that this might be caused by an incomplete integration of new cells into the circuitry of the spinal cord as the microenvironment caused by the injury favors the production of motor neurons over sensory afferent neurons [26].

## Discussion of studies that reported no neurogenic effects of PTBP1 knockdown

Successful conversions of glial cells to functional neurons hold huge promise in this field; however, several investigations (Table 3**)** that used more stringent lineage-tracing methodologies were either unable to detect new neurons following PTBP1 knockout/knockdown or unable to trace the origin of these new neurons to astrocytes [28–30, 63–65]. This discrepancy in results has sparked discussions in the field of regenerative medicine around current lineage tracing methodologies and strategies.

**Table 3:** Studies reporting lack of glia-to-neuron conversion following PTBP1 knockdown

Research group & reference	Intended target cell population	Intended CNS region	Animal model(s)	Targeting vector(s)	Lineage tracing	Detection method	Target sequence(s)	Improvements in animal model?
C.L. Zhang (Wang et al. (2021)) [30]	Astrocytes	Striatum	Young WT mice	AAV2/5-shPtbp1 (same sequence as Qian et al. (2020))	*Aldh1l1-CreER*^*T2*^*;R26R-YFP and mGfap-Cre;R26R-YFP*	Lineage reporter and viral reporter	5ʹ-GGGTGAAGATCCTGTTCAATA-3ʹ (exon 10 of mouse *Ptbp1*)	Not done – no generation of new neurons in vivo
				AAV2/5-LSL-shPtbp1 (same sequence as Qian et al. 2020))	*Aldh1l1-CreER*^*T2*^*;R26R-YFP and mGfap-Cre;R26R-YFP*	Lineage reporter and Cre-dependent viral reporter	5ʹ-GGGTGAAGATCCTGTTCAATA-3ʹ (exon 10 of mouse *Ptbp1*)	Not done – no generation of new neurons in vivo
				AAV2/2-LSL-shPtbp1 (vector from Qian et al. 2020))	*mGfap-Cre;R26R-YFP*	Lineage reporter and Cre-dependent viral reporter	5ʹ-GGGTGAAGATCCTGTTCAATA-3ʹ(exon 10 of mouse *Ptbp1*)	Not done – no generation of new neurons in vivo
				AAV2/5-LSL-shPtbp1 (vector from Qian et al. 2020))	*mGfap-Cre;R26R-YFP*	Lineage reporter and Cre-dependent viral reporter	5ʹ-GGGTGAAGATCCTGTTCAATA-3ʹ(exon 10 of mouse *Ptbp1*)	Not done – no generation of new neurons in vivo
				AAV2/2-LSL-shPtbp1 (virus from Qian et al. (2020))	*mGfap-Cre;R26R-YFP*	Lineage reporter and Cre-dependent viral reporter	5ʹ-GGGTGAAGATCCTGTTCAATA-3ʹ(exon 10 of mouse *Ptbp1*)	Not done – no generation of new neurons in vivo
				AAV2/PHP.eB-CRISPR-CasRX (vector from Zhou et al. (2020))	*mGfap-Cre;R26R-YFP*	Lineage reporter and viral reporter	5ʹ-GGGTGAAGATCCTGTTCAATA-3ʹ(exon 10 of mouse *Ptbp1*)	Not done – no generation of new neurons in vivo
S. Blackshaw (Hoang et al. (2023)) [63]	Astrocytes	Striatum	PD (6-OHDA)	Astrocyte-specific gene deletion	*Aldh1l1-CreER*^*T2*^*;Sun1- GFP*^*lox/lox*^*; Ptbp1*^*lox/lox*^	Viral reporter	N/A	No generation of new neurons in vivo
		Cortex	PD (6-OHDA)	Astrocyte-specific gene deletion	*Aldh1l1-CreER*^*T2*^*;Sun1- GFP*^*lox/lox*^*; Ptbp1*^*lox/lox*^	Viral reporter	N/A	No generation of new neurons in vivo
		SNpc	PD (6-OHDA)	Astrocyte-specific gene deletion	*Aldh1l1-CreER*^*T2*^*;Sun1- GFP*^*lox/lox*^*; Ptbp1*^*lox/lox*^	Viral Cre-induced reporter	N/A	No generation of new neurons in vivo
S. Blackshaw (Hoang et al. (2022)) [28]	Müller glia	Retina	NMDA injured mice	Müller glia-specific gene deletion	*Glast-CreER*^*T2*^*;Sun1-* *GFP*^*lox/lox*^*;Ptbp1*^*lox/lox*^	Viral Cre-induced reporter	N/A	Yes but unlikely to have resulted from *Ptbp1 *loss of function
B.L. Davidson (Leib et al. (2022)) [64]	Astrocytes	Striatum, cortex and hippocampus	Not done	AAV2/1-miPtbp1	*Aldh1l1-CreER*^*T2*^*;R26R-tdTomato*	Lineage reporter	5ʹ -CTCAATGTCAAGTACAACAAT-3’ (exon 7 of mouse *Ptbp1*)	No generation of new neurons in vivo
M. Li (Chen et al. (2022)) [29]	Astrocytes	Striatum	PD (6-OHDA)	AAV2/5-shPtbp1	*Aldh1l1-CreER*^*T2*^*;rpl22*^lsl-^^HA^	Lineage reporter and viral reporter	5ʹ-GGGTGAAGATCCTGTTCAATA-3ʹ (exon 10 of mouse *Ptbp1*)	No generation of new neurons in vivo
		SNpc	PD (6-OHDA)	Anti-*Ptbp1* ASO	*Aldh1l1-CreER*^*T2*^*;rpl22*^*lsl-HA*^	Lineage reporter and viral reporter	5ʹ-GTGGAAATATTGCTAGGCAC-3ʹ(same sequence as ASO2 in Maimon et al. (2021) targeting 5ʹ UTR of mouse *Ptbp1*)	No generation of new neurons in vivo
B. Chen (Xie et al. (2022)) [65]	Müller glia	Retina	NMDA-injured mice	AAV-CMV-LSL-RFP-shPtbp1 (same vector as Qian et al. (2020))	*Glast-CreER*^*T2*^*;Sun1-* *GFP*^*lox/lox*^	Lineage reporter and Cre-dependent viral reporter	5ʹ-GGGTGAAGATCCTGTTCAATA-3ʹ (exon 10 of mouse Ptbp1)	No
				AAV-GFAP-CasRx-Ptbp1 (same vector as Zhou et al. (2020))	*Glast-CreER*^*T2*^*;Sun1-* *GFP*^*lox/lox*^	Lineage reporter and viral reporter	Same guide RNA sequences as Zhou et al. (2020).	No
Y. Zhao (Guo et al. 2022) [76]	Astrocytes	Hippocampus	AD (5xFAD and PS19 mice), WT mice	AAV2/9-shPtbp1	Not done	Viral reporter	5ʹ-GGGTGAAGATCCTGTTCAATA-3 (Same sequence as Qian et al. (2020) targeting exon 10 of mouse *Ptbp1*)	No
K. Fang (Yang et al. 2023) [66]	Astrocytes	Striatum	Mouse WT	AAV-PHP.eB-Cas13X-NLS-HA-sgPtbp1	AAV-GFAP::tdTomato-WPRE	Lineage reporter	Vector sequnces provided in methods and supplemntary information	No

Table adapted from Wang & Zhang (2024) [62]. 6-OHDA, 6-hydroxydopamine; AAV, adeno-associated virus; ASO, antisense oligonucleotide; NMDA, N-methyl-*d*-aspartate; miRNA, micro-RNA; SNpc, substantia nigra pars compacta; WT, wild type

In testing the CRISPR/CasRX system that was reported to efficiently convert striatal astrocytes into dopaminergic neurons [25, 27], Wang et al. (2021) used *mGfap-Cre;R26R-YFP* mice instead of wild-type mice, allowing for a stringent lineage-tracing method of striatal astrocytes [30]. At a similar time point post-infection, PTBP1 expression was found only mildly reduced by CRISPR/CasRX, and ~ 22% of cells that were infected by the CasRX system were positive for the neuronal nuclear antigen marker (NeuN). However, none of these neuronal-marker-positive cells could be traced to the striatal astrocyte origin [30].

Next, Wang et al. (2021) tested the shPTBP1 system and the same vectors employed by Qian et al. (2020) using several AAV serotypes. Although they confirmed efficient knockdown of endogenous PTBP1 in striatal astrocytes, ~ 33% of cells that were NeuN^+^ were unable to be traced back to astrocytes in either *Aldh1l1-CreER*^*T2*^*;R26R-YFP* or *mGfap-Cre;R26R-YFP* mice [30]. The authors thus concluded that the reported new neurons seen in the initial studies [25, 27] were endogenous neurons that had been mistakenly reported as neurons generated from astrocyte reprogramming due to the leakage of AAV-based Cre recombination systems into endogenous neurons. Some of the authors of the original Zhou et al. (2020) study [27] have admitted it was the case in a new study [66]. A possible explanation for the discrepancy could be that the astrocyte-to-neuron conversion is inhibited by the lineage tracing systems applied. However, successful astrocyte-to-neuron conversions have been reported after manipulating either SOX2 or NEUROD1 expression in mice using the same astrocyte-labelling systems [67–70].

This brings up another potential confounding factor to astrocyte-to-neuron conversion – the AAV dosage employed. As reported by Xiang et al. (2021) [70], using a lower in vivo AAV dosage (10^10^–10^12^ gc/ml, 1 µl), glial cells could be reprogrammed through NEUROD1 overexpression, while at a higher dosage (2 × 10^13^ gc/ml, 1 µl), no new neurons could be traced back to astrocytes [30]. This is speculated that a higher amount of AAV could somehow inhibit astrocyte-to-neuron conversion due to cell stress or possibly cell death in reprogramming cells and promote unspecific labelling of endogenous neurons without affecting their survival. Nevertheless, following a “safe” dose of AAV-shPtbp1 (1 × 10^12^ gc/ml) injection into either the substantia nigra (1 μl) or striatum (2 μl), PTBP1 repression did not induce any new neurons that could be traced to the astrocyte lineage in an astrocyte-labelled 6-OHDA mouse model [29]. Therefore, whether AAV toxicity contributes to the discrepancy may warrant further investigations and more details on the discussions around this topic can be checked in journal correspondences [71, 72].

In another study, no neurons were generated from astrocytes and no therapeutic effect was observed in the 6-OHDA mouse model after CSF injection of an anti-PTBP1 ASO [29]. While the reported lack of astrocyte-originating neurons following PTBP1 suppression is very convincing, interestingly, the same ASO sequence targeting the 3′ UTR of mouse *Ptbp1* was reported to induce hippocampal neurogenesis in AD mouse models after intrathecal injection [24].

It is possible that differences in PTBP1-targeting ASO sequences, the chemical and backbone modifications of those ASOs, or the ASO secondary structures may result in distinct actions in different neuronal microenvironments depending on where an ASO is administered [73, 74]. A study in BioRxiv as a preprint explored this possibility [75], however, the data investigating these ASOs were excluded in their final peer-reviewed and published article [76]. Additionally, the time of administration after injury may also confound the evaluation of reprogramming outcomes. For example, early treatments post-injury may produce a misleading impression of successful reprogramming, as the prevention of neuronal loss may be caused by any unrevealed neuroprotective mechanisms. However, studies listed in Tables 2 and 3 performed experimentations at very similar timepoints in mice of similar age; thus, there would be other explanations for the contradictory results in the literature.

Several lines of evidence have been presented against studies reporting no effect of PTBP1-knockdown on glial cells, including 1) transient PTBP1 reduction following intra-cerebroventricular injection of ASO-PTBP1 resulted in the enhancement of new neurons in aged mice and in organoids; 2) the induced neurons matured morphologically in a time-dependent manner, rather than being already mature as one would expect in the case of reporter leakage; and 3) transient PTBP1 knockdown resulted in alleviation of symptoms in several aged disease mice from independent groups [24–27, 77, 78]. It is speculated that a reduction of anti-inflammatory cytokines or an improvement of glial microenvironment could contribute to therapeutic effects after the suppression of PTBP1. Many other possible roles and pathways that may be implicated in the regeneration of a damaged neuronal population following PTBP1 suppression have recently been discussed by Fu et al. [17] and Wang et al. [62]. However, this ongoing debate still leaves several outstanding questions to be addressed:

1. What is the determinant factor for the observed therapeutic effects following PTBP1 suppression if it was not directly related to astrocyte-to-neuron conversion? Pathway investigations are warranted as the role of PTBP1 is yet to be fully defined [79]. These may provide key insights into the clinical application of PTBP1 downregulation for the treatment of neurodegenerative diseases and clarify the controversies in the literature.
2. What is the cellular origin of the induced neurons? Were they derived from mature astrocytes, migratory radial glial cells, or rather a yet-to-be-defined glial population whose cell fate is affected by PTBP1 suppression?

Emerging evidence from a recent study supports the idea that PTBP1 suppression may induce neurogenesis through conversion of a different population of glial cells to astrocytes [77]. Through genetic barcoding and multiplexed error-robust fluorescence in situ hybridization, a high-throughput single-cell transcriptomics technique, researchers found that transient downregulation of PTBP1 induces re-activation of neurogenesis in developmentally active neurogenic niches in the dentate gyrus and subventricular zone of adult mice, leading to the generation of immature neurons [77, 80]. Two weeks after intracerebroventricular injection of an anti-*Ptbp1* ASO in one-year-old mice, *Ptbp1* RNA levels were suppressed by ~ 50% across the brain, with the highest knockdown level in the hippocampus and subventricular zone, which encompasses a large array of cells including astrocytes, neurons, choroid plexus and ependymal cells [77]. However, the RE1-silencing transcription factor (REST) complex, which is a repressive transcription factor that represses expression of a large set of neuronal genes in non-neuronal cells [58, 81, 82], was only reduced in choroid plexus cells and ependymal cells lining the subventricular wall [77], a region of neuronal precursors for brain repair [83]. Subsequently, the suppression of PTBP1 converted those cells into GABAergic inhibitory neurons, followed by progression through steps that mimicked neurogenesis [77].

However, not investigated in the above-mentioned studies, astrocytes in the subventricular zone were found to behave as pluripotent stem cells and could migrate to injured brain sites along with neuroblasts, participating in the generation of new neurons [84–90] and the formation of initial functional circuitry in spinal cord repair [88]. Nevertheless, there is limited research on the stability of the functional integration of these new neurons and how migrating astrocytes or precursor neurons might facilitate functional recovery in other disease models. This raises important questions regarding the neurogenic capabilities of astrocytes in other brain regions, as investigated in studies listed in Tables 2 and 3. Additionally, activated or reactive astrocytes share many features with radial glial cells [91], the latter of which are considered to be a glial cell lineage and serve as neurogenic progenitor cells [92–94]. However, this does not imply that all astrocytes have neurogenic potential since this heterogeneous group of glial cells display a number of distinct molecular, morphological, and functional signatures both between and within brain regions [95].

## Astrocyte heterogeneity providing insights into neuro-regeneration

Emerging spatial transcriptomics evidence supports the view that astrocytes need to be reclassified. The traditional binary categorization classifies astrocytes as being highly homogeneous cells that belong to two broad categories: fibrous astrocytes in white matter or protoplasmic astrocytes in grey matter [96, 97]. Under disease conditions resulting in insults to the CNS, resting astrocytes may become reactive in a process known as reactive astrogliosis, whereby astrocytes take on altered morphologies and become pro-inflammatory (A1 astrocytes) or neuroprotective (A2 astrocytes) according to their gene expression profiles [98]. Through single-cell, large-area spatial transcriptomics, regionally specific subtypes of astrocytes with distinct cell identities and cellular functions have been identified across the cortex and hippocampus of adult mice (Table 4) [99]. Cluster-analysis and spatial mapping revealed that astrocytes in different brain regions not only share genes for common astrocyte processes [100], but also express distinct genes depending on their anatomical location and function (Table 4).

**Table 4:** Currently redefined astrocyte subtypes and characteristics

Astrocyte type	Identity	Distribution	Characteristic genes	Defining roles
AST1	Mature astrocyte	Dominant subtype in hippocampus and subpial layer, spread throughout cortex	High expression of *Gfap* and *Agt*	Synaptogenesis, synaptic plasticity, glutamatergic neurotransmission
AST2	Mature astrocyte	Uniformly distributed across cortical layers	High expression of *Unc13c*, absent expression of *Agt*	Glutamatergic neurotransmission
AST3	Mature astrocyte	Dominant subtype in layer 6 of cortex	Expression of* Agt, *absent expression of* Unc13c *and* Gfap*	GABAergic neurotransmission
AST4	Hippocampal neural stem cells/progenitor astrocyte	High levels in dentate gyrus of hippocampus, predominantly found in subgranular layer of hippocampus	High expression of *Frzb, Ascl1, Slc1a3*, *Sirt2, Sept2,* and* Emp2*	Mitosis and cell cycle control; Transcriptional regulation; Neurogenesis and neuronal differentiation
AST5	Intermediate progenitor astrocyte	High in cortical layers 2/3, and 5 Dominant in subpial layer, stratum lacunosum-moleculare and dentate gyrus of hippocampus	High expression of *Frzb, Ascl1, Slc1a3*, *Sirt2, Sept2, *and *Emp2*	Mitosis and cell cycle control; Glucose metabolism; Energy production; Smallest proportion of astrocyte types (1.4%)

Note: astrocyte subtypes are proposed by Batiuk et al. (2020) [99]

In support of these findings, bulk RNA sequencing has revealed that astrocytes and neurons share region-specific transcriptional and epigenetic signatures that facilitate the conversion of astrocytes to the desired neuronal type in reprogramming strategies [101]. This may explain why primarily striatal astrocytes were reported to be reprogrammed to striatal neurons in vivo following stereotaxic injections of ASOs or AAVs suppressing PTBP1 into mouse substantia nigra [25], and why reactive spinal cord astrocytes were converted mostly to motor neurons in spinal cord injury mice after local injections of anti-PTBP1 ASO [26]. Although the latter study did not reveal which astrocyte subsets were targeted by PTBP1 ASO treatments [26], it would be interesting to investigate whether ventral horn astrocytes were preferentially targeted to contribute to the generation of motor neurons, as ventral horn astrocyte populations and motor neurons have shared lineages. This could further support the hypothesis that regional astrocytes can transform into their surrounding neurons because of their shared genetic signatures [101] and the capabilities of astrocytes to maintain and support neuronal growth in a region-specific manner [102, 103]. In support of that, intracerebroventricular administration of an anti-PTBP1 ASO resulted in the highest knockdown level of glial PTBP1 in the hippocampus and generation of predominantly hippocampal neurons [24]. However, the mechanisms underlying the preferential distributions of the anti-PTBP1 approaches after local administrations and why other brain regions were not affected by PTBP1 suppression warrants further investigations.

Furthermore, using spatial transcriptomic analysis, researchers found that astrocytes organize themselves in a gradient-layer pattern throughout the cortex in a different manner compared to the classic cortical layers of neurons in the mammalian brain [31]. Even within the specialized astrocyte populations, another layer of diversity exists: they are organized into two categories as to whether they possess inhibitory or excitatory synapse properties. This then prompts the intriguing question of whether layer-specific reprogramming is driven by cell-intrinsic mechanisms, specific migration, or environmental cues. Interestingly, astrocytes located in upper versus lower layers differ not only in their morphology but also in gene expression profiles [104]. Surrounding neurons may play a key role in this process, as demonstrated in the cerebellum, where neuron-released sonic hedgehog influenced local astrocyte transcriptional activity [105]. This supports the hypothesis that the layer-dependent differences in cortical astrocytes might affect the outcome of reprogramming in terms of neuronal subtype identity. However, further studies are needed to characterize the identity of astrocytes and make comparisons between different astrocyte subtypes in other CNS regions including the brainstem and spinal cord for the development of effective and targeted therapies.

## Therapeutic modalities and delivery systems suitable for targeted astrocyte-to-neuron conversion

**Fig. 1:**
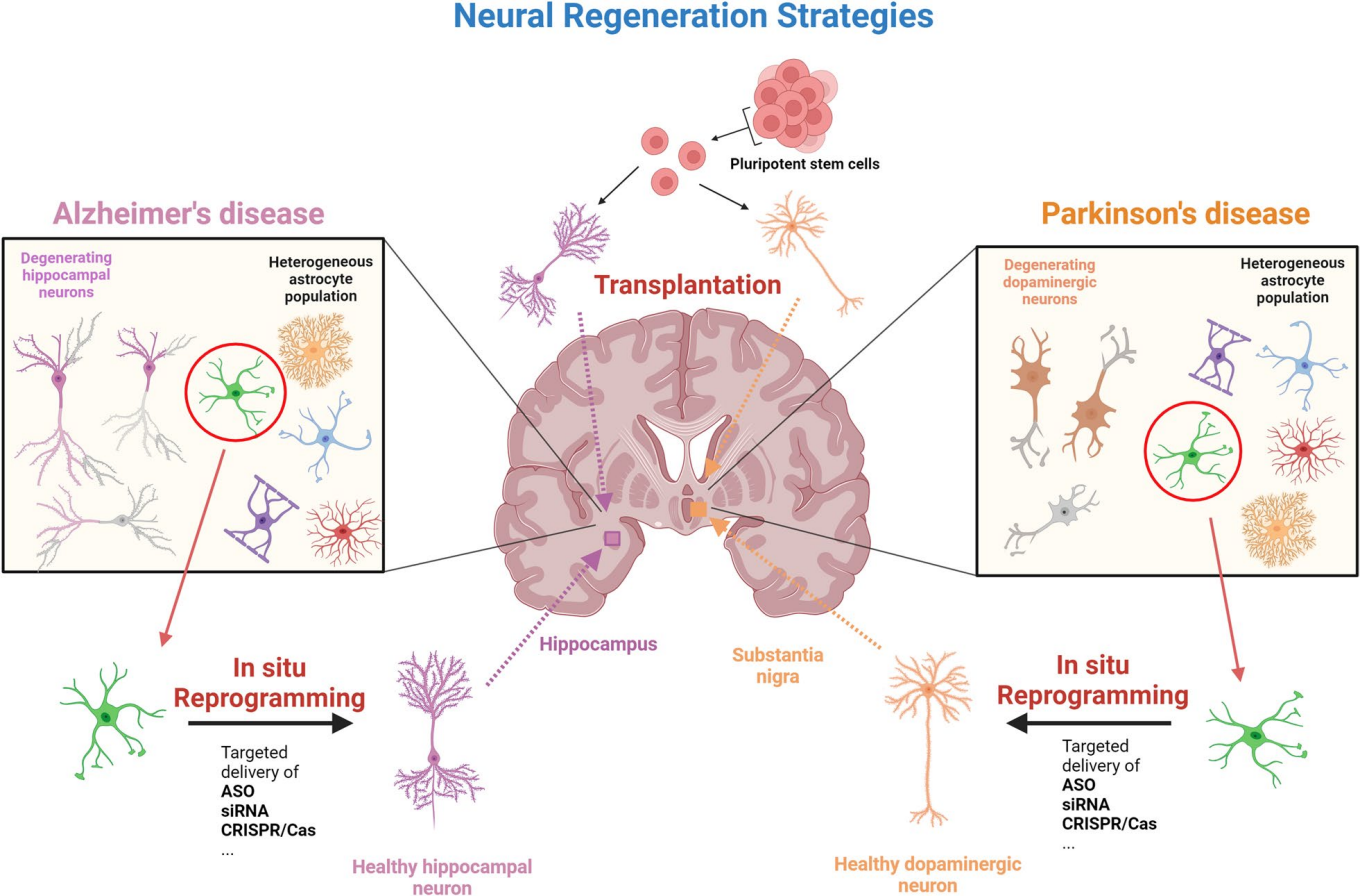
**Neural regeneration strategies to treat Alzheimer’s (AD) or Parkinson’s disease (AD).** Stem cell-based transplantation strategies have been trialed to treat patients with AD or PD. The reprogramming of astrocytes that share region-specific genetic, epigenetic, and molecular signatures with the regional neurons may serve as an alternative approach to generating new neurons in situ as a promising treatment option for AD and PD, once the most appropriate therapeutics including antisense oligonucleotides (ASOs), small interfering RNAs (siRNAs), and CRISPR/Cas and targeted delivery systems are developed. Figure was created with BioRender.com

The specific transcriptomic and epigenomic signatures shared by astrocytes and neurons and the neurogenic potential of certain astrocyte subtypes have indicated the promise of converting regional astrocytes into corresponding neuronal types as a potential and effective neuro-regenerative treatment. Certain subtypes of astrocyte that are near either hippocampal neurons or dopaminergic neurons may potentially be transdifferentiated into healthy and functional hippocampal neurons or dopaminergic neurons to replenish the lost neurons in AD or PD patients (Fig. 1). However, to achieve this, highly efficient therapeutic and delivery systems are required.

### ASOs

ASO therapeutics is one of the emerging techniques that have demonstrated great potential as therapeutics for many different diseases. ASOs are short (usually 15–30 nucleotides in length) synthetic nucleic acid analogues that can be designed to bind to their RNA targets with high specificity through Watson–Crick base pairing [106, 107]. Once bound to targeted regions of an RNA transcript, ASOs building on different chemical modifications can affect gene expression through a variety of mechanisms, such as steric-blocking of the cis-acting RNA regulatory motifs to physically interrupt the progression of premature messenger RNA splicing [107], which can result in an increase or decrease in the expression of targeted genes through different mechanisms. For example, splice-switching ASOs can be utilized to excise an out-of-frame exon which creates a premature stop codon and subsequently results in nonsense-mediated decay of the target transcripts. In addition, RNase-H-inducing ASOs, once specifically binding to target mRNAs, form DNA:RNA hybrids and recruit RNase-H, which subsequently cleaves the mRNAs. Details on how antisense technology can be utilized to manipulate gene expression and their potential therapeutic applications in CNS disorders and other diseases can be found in reviews [108–110]. Over 10 ASO drugs have now been approved for clinical use for genetic subgroups of several neurological and CNS diseases including Duchenne muscular dystrophy, spinal muscular atrophy, and amyotrophic lateral sclerosis. Meanwhile, substantial investigations are also ongoing to develop ASO therapeutics for PD and AD, including ASOs to suppress PTBP1 that have been tried in several studies both in vitro and in animal models [24, 25].

### siRNAs

The field of siRNA therapeutics has progressed rapidly. It only took 20 years from the discovery of the RNA interfering (RNAi) technique to the development of Patisiran, the first siRNA drug to acquire FDA approval [111]. In recent years, a few other drugs including Vutrisiran, Givosiran, and Lumasiran have been approved for several indications. Although all the current approved siRNA therapeutics have the liver as the primary target tissue, the conjugation of siRNAs to lipophilic 2′-O-hexadecyle has expanded RNAi therapeutics to the CNS with safe, potent, and durable silencing of targets in mouse and nonhuman primate models of AD and amyotrophic lateral sclerosis as reported by Alnylam Pharmaceuticals [112]. Unlike the single-stranded ASOs, siRNAs are synthetic double-stranded RNAs of approximately 20–25 base pairs. Like ASOs, synthetic siRNAs require essential chemical modifications to promote their stability and enhance their activities to specifically and efficiently degrade target mRNAs by exploiting the RNAi pathway [113]. Promising data have been reported from several different siRNA therapeutic strategies that tried to target surrogate proteins including alpha-synuclein, amyloid-β, and huntingtin which are involved in proteinopathies in neurodegenerative disorders including AD, PD, and Huntington’s disease [114]. However, no siRNA treatments have yet been approved for clinical use for CNS disorders. It is likely that improvements in siRNA design, chemical modifications, and development of siRNA delivery systems including the 2′-O-hexadecyle conjugate or accessory oligonucleotide-siRNA duplexes [115] will likely lead to successful translations of siRNA therapeutics for CNS diseases.

### CRISPR/CRISPR-associated protein 9 (Cas) gene editing techniques

The discovery of gene editing techniques, including zinc finger nuclease in the late 1900s, has enabled highly efficient and targeted gene engineering. However, gene editing therapeutics has only shown some promise since 2012 when the CRISPR/Cas9 system was developed as a genome editing tool [116, 117]. Naturally evolved in bacteria as a defense mechanism again viruses, the CRISPR/Cas9 enables precise editing and repair of genes by the Cas9 endonuclease to induce double-stranded breaks at target DNA sequences. These breaks can then be repaired through homology-directed repair, facilitating the insertion of new genes, or allowing for base editing when an appropriate repair template is provided. Although there are still concerns over the CRISPR gene editing technique including immunogenicity, off-target effects, and ethical issues, significant therapeutic benefits in eliminating severe vaso-occlusive crises as seen in clinical trials have led to FDA approvals of two independent CRISPR/Cas9 gene editing therapies (Casgevy and Lyfgenia) to treat patients with severe sickle cell disease [118]. No evidence of genotoxicity was reported from these trials [119]; however, patients may still prefer to wait until the community has more experience with this kind of therapy and uncertainties of the long-term safety are resolved. CRISPR/Cas-mediated single-base editing and prime editing systems are expected to have less safety concerns since these novel techniques produce precise base changes instead of introducing double stranded breaks [120]; however, severe adverse events, including a death in the clinical trial, have drawn criticism despite significant therapeutic efficacy observed using base-editing [121]. Nevertheless, CRISPR/Cas-associated gene editing therapies still hold considerable promise to treat diseases including PD and AD, or to improve current therapeutic options such as the trial of base-edited CAR-T cells for a rare subtype of leukemia [122].

### Targeted CNS delivery strategies

Gene therapies including ASOs, siRNAs, and CRISPR/Cas-associated gene editing system have shown significant promise in developing novel disease-modifying treatments for neurodegenerative disorders including PD and AD. However, delivering therapeutic compounds across the blood–brain barrier (BBB) is still the main challenge hampering the translation of CNS drugs. Poor BBB permeation of systemically delivered un-conjugated siRNAs and ASOs has been reported in the literature [123, 124] and on average less than 1% of ASOs can reach RNA targets in the brain [125, 126]. Moreover, to achieve targeted astrocyte-to-neuron transdifferentiation, a much more precise and potent system is required to allow brain region/layer-specific and cell subtype-specific delivery of therapeutics to a particular population of astrocytes.

Improvements in nucleic acid chemistries or chemical modifications, and the development of novel delivery systems, are bringing gene therapies for CNS disorders closer to clinical applications. Tricyclo-DNA, a comparatively new ASO chemistry, has been reported to moderately reach the CNS after systemic administration and induce a promising but low level of target engagement in mouse hippocampus and cortex, while other chemistries demonstrated limited activities [127]. However, further refinement may be needed for this alternative ASO chemistry due to the potential toxicity as seen in tricyclo-DNA-treated mice [128]. Safe, potent, and long-term gene silencing effects have been reported for local administrations of divalent or multiple-valent siRNAs that are composed of more than one fully chemically modified siRNAs in several tissue types in animal models [129–131].

To achieve effective CNS or astrocyte-targeted delivery through systemic administrations, conjugating ASOs or siRNAs to various moieties, including nanoparticles, antibodies, peptides, exosomes, and lipids has demonstrated some promise. For example, lipid nanoparticles formulated from adenosine-conjugated lipids and an ionizable lipid have enabled specific uptake of an siRNA targeting toll-like receptor 4 (TLR4) by astrocytes near damaged brain tissue in a traumatic brain injury mouse model which resulted in a substantial knockdown of both TLR4 mRNA and protein after intravenous injections [132]. In addition, an ASO constructed with an A1 astrocyte-targeted peptide achieved specific gene silencing in hippocampal astrocytes in a mouse model through tail vein injections [133].

The progress in nanoparticle, cell-penetrating peptide, and lipid delivery strategies can also be applied to CRISPR/Cas gene editing system as non-viral vectors [134, 135]; however, viral vectors including AAV-9 as used in Zolgesma, a gene therapy for spinal muscular atrophy, and AAV-rh10 are commonly used capsids for CNS delivery [136, 137]. Several AAV serotypes are capable of transducing astrocytes. For example, AAV-4 was shown to transduce astrocytes in the subventricular zone after intracerebroventricular injections [138] and AAV-9 could efficiently transduce astroglial cells through intravenous administrations [136, 139]. To further improve BBB penetration and improve cell specific delivery, a recent study has found that chimeric AAV capsids show an improved and preferential transduction of astrocytes and neurons. A combination of amino acids from regions 413–496 of AAV-rh10 and 538–598 of AAV-3B/LK03 delivered higher amounts of vector genomic DNA to astrocytes compared to standard AAV-9 and AAV-rh10 serotypes [140]. Progress in this area has favored the application of CRISPR/Cas gene editing techniques for neurodegenerative disease as well as astrocyte-to-neuron conversions.

## Conclusions and future perspectives

The prospect of targeted conversion of glial cells into functional neurons has shown significant potential as a neuro-regenerative strategy to replenish lost neurons in CNS conditions including PD and AD. Further discussions and investigations will be required to ascertain with greater certainty the origin of newly generated neurons using more stringent lineage tracing methods or using novel live cell imaging methods to capture astrocyte-to-neuron intermediate states. A more detailed understanding of the neurobiology of glial cells, and in particular of the regional heterogeneity of astrocyte populations and their specific functions, will benefit the broader field of neuroscience and provide valuable insights into the debate over astrocyte-to-neuron transdifferentiation, paving the way for the development of novel neuro-regeneration therapeutics for AD, PD, and other neurodegenerative disorders. However, the consequences of long-term astrocyte-to-neuron conversions, even if highly specific delivery systems are developed to target particular subsets of astrocytes in defined CNS regions, warrant further and long-term investigations.

## Data Availability

Not applicable.

## Abbreviations

AD: Alzheimer’s disease
PD: Parkinson’s disease
PTBP1: Polypyrimidine tract-binding protein 1
CNS: Central nervous system
ESCs: Embryonic stem cells
iPSCs: Induced pluripotent stem cells
MSCs: Mesenchymal stem cells
shRNA: Short hairpin RNAs
ASOs: Antisense oligonucleotides
6-OHDA: 6-Hydroxydopamine
WT: Wild type
miRNA: Micro-RNA
CSF: Cerebrospinal fluid
mRNA: Messenger RNA
siRNAs: Small interfering RNAs
RNAi: RNA interfering
BBB: Blood–brain barrier
TLR4: Toll-like receptor 4

## References

[1] Feigin VL, Vos T, Nichols E, Owolabi MO, Carroll WM, Dichgans M, et al. The global burden of neurological disorders: translating evidence into policy. Lancet Neurol. 2020;19(3):255–65. 31813850 10.1016/S1474-4422(19)30411-9 PMC9945815

[2] Xu W, Chen L, Cai G, Gao M, Chen Y, Pu J, et al. Diagnosis of parkinson’s disease via the metabolic fingerprint in saliva by deep learning. Small Methods. 2023;7(7):2300285. 10.1002/smtd.202300285 37236160

[3] Misiura MB, Butts B, Hammerschlag B, Munkombwe C, Bird A, Fyffe M, et al. Intersectionality in Alzheimer’s disease: the role of female sex and Black American race in the development and prevalence of Alzheimer’s disease. Neurotherapeutics. 2023;20(4):1019–36. 37490246 10.1007/s13311-023-01408-x PMC10457280

[4] Scheltens P, De Strooper B, Kivipelto M, Holstege H, Chételat G, Teunissen CE, et al. Alzheimer’s disease. Lancet. 2021;397(10284):1577–90. 33667416 10.1016/S0140-6736(20)32205-4 PMC8354300

[5] Church FC. Treatment options for motor and non-motor symptoms of Parkinson’s disease. Biomolecules. 2021;11(4):612. 33924103 10.3390/biom11040612 PMC8074325

[6] Pang SY-Y, Ho PW-L, Liu H-F, Leung C-T, Li L, Chang EES, et al. The interplay of aging, genetics and environmental factors in the pathogenesis of Parkinson’s disease. Transl Neurodegener. 2019;8:1–11. 31428316 10.1186/s40035-019-0165-9 PMC6696688

[7] Wilson J, Alcock L, Yarnall AJ, Lord S, Lawson RA, Morris R, et al. Gait progression over 6 years in Parkinson’s disease: effects of age, medication, and pathology. Front Aging Neurosci. 2020;12:577435. 33192470 10.3389/fnagi.2020.577435 PMC7593770

[8] Lampropoulos IC, Malli F, Sinani O, Gourgoulianis KI, Xiromerisiou G. Worldwide trends in mortality related to parkinson’s disease in the period of 1994–2019: analysis of vital registration data from the WHO Mortality Database. Front Neurol. 2022;13:956440. 36267881 10.3389/fneur.2022.956440 PMC9576872

[9] Rajan KB, Weuve J, Barnes LL, McAninch EA, Wilson RS, Evans DA. Population estimate of people with clinical Alzheimer’s disease and mild cognitive impairment in the United States (2020–2060). Alzheimers Dement. 2021;17(12):1966–75. 34043283 10.1002/alz.12362 PMC9013315

[10] Haque R, Alam K, Gow J, Neville C. Changes in the prevalence of dementia in Australia and its association with geographic remoteness. PLoS One. 2023;18(8):e0289505. 37531396 10.1371/journal.pone.0289505 PMC10395934

[11] Kepchia D, Huang L, Dargusch R, Rissman RA, Shokhirev MN, Fischer W, et al. Diverse proteins aggregate in mild cognitive impairment and Alzheimer’s disease brain. Alzheimers Res Ther. 2020;12:1–20. 10.1186/s13195-020-00641-2 PMC7305608 32560738

[12] Planche V, Manjon JV, Mansencal B, Lanuza E, Tourdias T, Catheline G, et al. Structural progression of Alzheimer’s disease over decades: the MRI staging scheme. Brain Commun. 2022;4(3):109. 10.1093/braincomms/fcac109 PMC9113086 35592489

[13] Trejo-Lopez JA, Yachnis AT, Prokop S. Neuropathology of Alzheimer’s disease. Neurotherapeutics. 2023;19(1):173–85. 10.1007/s13311-021-01146-y PMC9130398 34729690

[14] Cajal SR. Degeneration & regeneration of the nervous system. Oxford University Press, Humphrey Milford; 1928

[15] Mousaei Ghasroldasht M, Seok J, Park H-S, Liakath Ali FB, Al-Hendy A. Stem cell therapy: from idea to clinical practice. Int J Mol Sci. 2022;23(5):2850. 35269990 10.3390/ijms23052850 PMC8911494

[16] Omole AE, Fakoya AOJ, Nnawuba KC, Haider KH. Common ethical considerations of human-induced pluripotent stem cell research. London: Springer; 2022. p. 1–17.

[17] Fu X-D, Mobley WC. Therapeutic potential of PTB inhibition through converting glial cells to neurons in the brain. Annu Rev Neurosci. 2023;46(1):145–65. 37428606 10.1146/annurev-neuro-083022-113120

[18] Talifu Z, Liu JY, Pan YZ, Ke H, Zhang CJ, Xu X, et al. In vivo astrocyte-to-neuron reprogramming for central nervous system regeneration: a narrative review. Neural Regen Res. 2023;18(4):750–5. 36204831 10.4103/1673-5374.353482 PMC9700087

[19] Su Z, Niu W, Liu ML, Zou Y, Zhang CL. In vivo conversion of astrocytes to neurons in the injured adult spinal cord. Nat Commun. 2014;5:3338. 24569435 10.1038/ncomms4338 PMC3966078

[20] Ma NX, Puls B, Chen G. Transcriptomic analyses of NeuroD1-mediated astrocyte-to-neuron conversion. Dev Neurobiol. 2022;82(5):375–91. 35606902 10.1002/dneu.22882 PMC9540770

[21] Puls B, Ding Y, Zhang F, Pan M, Lei Z, Pei Z, et al. Regeneration of functional neurons after spinal cord injury via in situ NeuroD1-mediated astrocyte-to-neuron conversion. Front Cell Dev Biol. 2020;8:591883. 33425896 10.3389/fcell.2020.591883 PMC7793709

[22] Tang Y, Wu Q, Gao M, Ryu E, Pei Z, Kissinger ST, et al. Restoration of visual function and cortical connectivity after ischemic injury through NeuroD1-mediated gene therapy. Front Cell Dev Biol. 2021;9:720078. 34490268 10.3389/fcell.2021.720078 PMC8416524

[23] Chen Y-C, Ma N-X, Pei Z-F, Wu Z, Do-Monte FH, Keefe S, et al. A NeuroD1 AAV-based gene therapy for functional brain repair after ischemic injury through in vivo astrocyte-to-neuron conversion. Mol Ther. 2020;28(1):217–34. 31551137 10.1016/j.ymthe.2019.09.003 PMC6952185

[24] Maimon R, Chillon-Marinas C, Snethlage CE, Singhal SM, McAlonis-Downes M, Ling K, et al. Therapeutically viable generation of neurons with antisense oligonucleotide suppression of PTB. Nat Neurosci. 2021;24(8):1089–99. 34083786 10.1038/s41593-021-00864-y PMC8338913

[25] Qian H, Kang X, Hu J, Zhang D, Liang Z, Meng F, et al. Reversing a model of Parkinson’s disease with in situ converted nigral neurons. Nature. 2020;582(7813):50–6. 32581380 10.1038/s41586-020-2388-4 PMC7521455

[26] Yang R-Y, Chai R, Pan J-Y, Bao J-Y, Xia P-H, Wang Y-K, et al. Knockdown of polypyrimidine tract binding protein facilitates motor function recovery after spinal cord injury. Neural Regen Res. 2023;18(2):396–403. 35900436 10.4103/1673-5374.346463 PMC9396513

[27] Zhou H, Su J, Hu X, Zhou C, Li H, Chen Z, et al. Glia-to-neuron conversion by CRISPR-CasRx alleviates symptoms of neurological disease in mice. Cell. 2020;181(3):590.e16-603.e16. 32272060 10.1016/j.cell.2020.03.024

[28] Hoang T, Kim DW, Appel H, Pannullo NA, Leavey P, Ozawa M, et al. Genetic loss of function of Ptbp1 does not induce glia-to-neuron conversion in retina. Cell Rep. 2022;39(11):110849. 35705053 10.1016/j.celrep.2022.110849 PMC9619396

[29] Chen W, Zheng Q, Huang Q, Ma S, Li M. Repressing PTBP1 fails to convert reactive astrocytes to dopaminergic neurons in a 6-hydroxydopamine mouse model of Parkinson’s disease. Elife. 2022;11:e75636. 35535997 10.7554/eLife.75636 PMC9208759

[30] Wang L-L, Serrano C, Zhong X, Ma S, Zou Y, Zhang C-L. Revisiting astrocyte to neuron conversion with lineage tracing in vivo. Cell. 2021;184(21):5465–8116. 34582787 10.1016/j.cell.2021.09.005 PMC8526404

[31] Bayraktar OA, Bartels T, Holmqvist S, Kleshchevnikov V, Martirosyan A, Polioudakis D, et al. Astrocyte layers in the mammalian cerebral cortex revealed by a single-cell in situ transcriptomic map. Nat Neurosci. 2020;23(4):500–9. 32203496 10.1038/s41593-020-0602-1 PMC7116562

[32] Hoang DM, Pham PT, Bach TQ, Ngo AT, Nguyen QT, Phan TT, et al. Stem cell-based therapy for human diseases. Signal Transduct Target Ther. 2022;7(1):1–41. 35933430 10.1038/s41392-022-01134-4 PMC9357075

[33] Shihabuddin LS, Aubert I. Stem cell transplantation for neurometabolic and neurodegenerative diseases. Neuropharmacology. 2010;58(6):845–54. 20036262 10.1016/j.neuropharm.2009.12.015

[34] Xian B, Huang B. The immune response of stem cells in subretinal transplantation. Stem Cell Res Ther. 2015;6:1–7. 26364954 10.1186/s13287-015-0167-1 PMC4568575

[35] Maherali N, Sridharan R, Xie W, Utikal J, Eminli S, Arnold K, et al. Directly reprogrammed fibroblasts show global epigenetic remodeling and widespread tissue contribution. Cell Stem Cell. 2007;1(1):55–70. 18371336 10.1016/j.stem.2007.05.014

[36] Pappas JJ, Yang PC. Human ESC vs. iPSC—pros and cons. J Cardiovasc Transl Res. 2008;1:96–9. 20559900 10.1007/s12265-008-9032-2

[37] Fan Y, Winanto NS-Y. Replacing what’s lost: a new era of stem cell therapy for Parkinson’s disease. Transl Neurodegener. 2020;9:1–10. 31911835 10.1186/s40035-019-0180-x PMC6945567

[38] Huang C-Y, Liu C-L, Ting C-Y, Chiu Y-T, Cheng Y-C, Nicholson MW, et al. Human iPSC banking: barriers and opportunities. J Biomed Sci. 2019;26:1–14. 31660969 10.1186/s12929-019-0578-x PMC6819403

[39] Bravery CA. Do human leukocyte antigen-typed cellular therapeutics based on induced pluripotent stem cells make commercial sense? Stem Cells Dev. 2015;24(1):1–10. 25244598 10.1089/scd.2014.0136

[40] Jacquet L, Stephenson E, Collins R, Patel H, Trussler J, Al-Bedaery R, et al. Strategy for the creation of clinical grade hESC line banks that HLA-match a target population. EMBO Mol Med. 2013;5(1):10–7. 23161805 10.1002/emmm.201201973 PMC3569650

[41] Ortuño-Costela MDC, Cerrada V, García-López M, Gallardo ME. The challenge of bringing iPSCs to the patient. Int J Mol Sci. 2019;20(24):6305. 31847153 10.3390/ijms20246305 PMC6940848

[42] Dulak J, Szade K, Szade A, Nowak W, Józkowicz A. Adult stem cells: hopes and hypes of regenerative medicine. Acta Biochim Pol. 2015;62(3):329–37. 26200199 10.18388/abp.2015_1023

[43] Zhao Q, Ren H, Han Z. Mesenchymal stem cells: Immunomodulatory capability and clinical potential in immune diseases. J Cell Immunother. 2016;2(1):3–20.

[44] Musiał-Wysocka A, Kot M, Majka M. The pros and cons of mesenchymal stem cell-based therapies. Cell Transplant. 2019;28(7):801–12. 31018669 10.1177/0963689719837897 PMC6719501

[45] Lalu MM, McIntyre L, Pugliese C, Fergusson D, Winston BW, Marshall JC, et al. Safety of cell therapy with mesenchymal stromal cells (safecell): a systematic review and meta-analysis of clinical trials. PLoS One. 2012;7(10):e47559. 23133515 10.1371/journal.pone.0047559 PMC3485008

[46] Berkowitz AL, Miller MB, Mir SA, Cagney D, Chavakula V, Guleria I, et al. Glioproliferative lesion of the spinal cord as a complication of “stem-cell tourism.” N Engl J Med. 2016;375(2):196–8. 27331440 10.1056/NEJMc1600188

[47] Shinagawa K, Kitadai Y, Tanaka M, Sumida T, Kodama M, Higashi Y, et al. Mesenchymal stem cells enhance growth and metastasis of colon cancer. Int J Cancer. 2010;127(10):2323–33. 20473928 10.1002/ijc.25440

[48] Hejrati N, Wong R, Khazaei M, Fehlings MG. How can clinical safety and efficacy concerns in stem cell therapy for spinal cord injury be overcome? Expert Opin Biol Ther. 2023;23(9):883–99. 37545020 10.1080/14712598.2023.2245321

[49] Wang Z. Assessing tumorigenicity in stem cell-derived therapeutic products: a critical step in safeguarding regenerative medicine. Bioengineering. 2023;10(7):857. 37508884 10.3390/bioengineering10070857 PMC10376867

[50] Bonaventura G, Munafò A, Bellanca CM, La Cognata V, Iemmolo R, Attaguile GA, et al. Stem cells: innovative therapeutic options for neurodegenerative diseases? Cells. 2021;10(8):1992. 34440761 10.3390/cells10081992 PMC8391848

[51] Vasan L, Park E, David LA, Fleming T, Schuurmans C. Direct neuronal reprogramming: bridging the gap between basic science and clinical application. Front Cell Dev Biol. 2021;9:681087. 34291049 10.3389/fcell.2021.681087 PMC8287587

[52] Kim SM, Flaßkamp H, Hermann A, Araúzo-Bravo MJ, Lee SC, Lee SH, et al. Direct conversion of mouse fibroblasts into induced neural stem cells. Nat Protoc. 2014;9(4):871–81. 24651499 10.1038/nprot.2014.056

[53] Dennison R, Usuga E, Chen H, Paul JZ, Arbelaez CA, Teng YD. Direct cell reprogramming and phenotypic conversion: an analysis of experimental attempts to transform astrocytes into neurons in adult animals. Cells. 2023;12(4):618. 36831283 10.3390/cells12040618 PMC9954435

[54] Lima Cunha D, Arno G, Corton M, Moosajee M. The spectrum of PAX6 mutations and genotype-phenotype correlations in the eye. Genes. 2019;10(12):1050. 31861090 10.3390/genes10121050 PMC6947179

[55] Heins N, Malatesta P, Cecconi F, Nakafuku M, Tucker KL, Hack MA, et al. Glial cells generate neurons: the role of the transcription factor Pax6. Nat Neurosci. 2002;5(4):308–15. 11896398 10.1038/nn828

[56] Harvey AR, Lovett SJ, Majda BT, Yoon JH, Wheeler LPG, Hodgetts SI. Neurotrophic factors for spinal cord repair: which, where, how and when to apply, and for what period of time? Brain Res. 2015;1619:36–71. 25451132 10.1016/j.brainres.2014.10.049

[57] Guo Z, Zhang L, Wu Z, Chen Y, Wang F, Chen G. In vivo direct reprogramming of reactive glial cells into functional neurons after brain injury and in an Alzheimer’s disease model. Cell Stem Cell. 2014;14(2):188–202. 24360883 10.1016/j.stem.2013.12.001 PMC3967760

[58] Xue Y, Ouyang K, Huang J, Zhou Y, Ouyang H, Li H, et al. Direct conversion of fibroblasts to neurons by reprogramming PTB-regulated microRNA circuits. Cell. 2013;152(1–2):82–96. 23313552 10.1016/j.cell.2012.11.045 PMC3552026

[59] Dai S, Wang C, Zhang C, Feng L, Zhang W, Zhou X, et al. PTB: not just a polypyrimidine tract-binding protein. J Cell Physiol. 2022;237(5):2357–73. 35288937 10.1002/jcp.30716

[60] Monzón-Casanova E, Matheson LS, Tabbada K, Zarnack K, Smith CW, Turner M. Polypyrimidine tract-binding proteins are essential for B cell development. Elife. 2020;9:53557. 10.7554/eLife.53557 PMC7058386 32081131

[61] Xue Y, Qian H, Hu J, Zhou B, Zhou Y, Hu X, et al. Sequential regulatory loops as key gatekeepers for neuronal reprogramming in human cells. Nat Neurosci. 2016;19(6):807–15. 27110916 10.1038/nn.4297 PMC4882254

[62] Wang L-L, Zhang C-L. Therapeutic potential of PTBP1 inhibition, if any, is not attributed to glia-to-neuron conversion. Annu Rev Neurosci. 2023;46(1):1–15. 36750409 10.1146/annurev-neuro-092822-083410 PMC10404630

[63] Hoang T, Kim DW, Appel H, Ozawa M, Zheng S, Kim J, et al. Ptbp1 deletion does not induce astrocyte-to-neuron conversion. Nature. 2023;618(7964):E1-e7. 37286658 10.1038/s41586-023-06066-9 PMC12327411

[64] Leib D, Chen YH, Monteys AM, Davidson BL. Limited astrocyte-to-neuron conversion in the mouse brain using NeuroD1 overexpression. Mol Ther. 2022;30(3):982–6. 35123657 10.1016/j.ymthe.2022.01.028 PMC8899704

[65] Xie Y, Zhou J, Chen B. Critical examination of Ptbp1-mediated glia-to-neuron conversion in the mouse retina. Cell Rep. 2022;39(11):110960. 35705044 10.1016/j.celrep.2022.110960 PMC9371382

[66] Yang G, Yan Z, Wu X, Zhang M, Xu C, Shi L, et al. Ptbp1 knockdown failed to induce astrocytes to neurons in vivo. Gene Ther. 2023;30(12):801–6. 36721028 10.1038/s41434-023-00382-5

[67] Su Z, Niu W, Liu M-L, Zou Y, Zhang C-L. In vivo conversion of astrocytes to neurons in the injured adult spinal cord. Nat Commun. 2014;5(1):3338. 24569435 10.1038/ncomms4338 PMC3966078

[68] Wang L-L, Su Z, Tai W, Zou Y, Xu X-M, Zhang C-L. The p53 pathway controls SOX2-mediated reprogramming in the adult mouse spinal cord. Cell Rep. 2016;17(3):891–903. 27732862 10.1016/j.celrep.2016.09.038 PMC5094368

[69] Tai W, Wu W, Wang L-L, Ni H, Chen C, Yang J, et al. In vivo reprogramming of NG2 glia enables adult neurogenesis and functional recovery following spinal cord injury. Cell Stem Cell. 2021;28(5):923e4-37e4. 33675690 10.1016/j.stem.2021.02.009 PMC8106641

[70] Xiang Z, Xu L, Liu M, Wang Q, Li W, Lei W, et al. Lineage tracing of direct astrocyte-to-neuron conversion in the mouse cortex. Neural Regen Res. 2021;16(4):750–6. 33063738 10.4103/1673-5374.295925 PMC8067918

[71] Chen G. In vivo confusion over in vivo conversion. Mol Ther. 2021;29(11):3097–8. 34699780 10.1016/j.ymthe.2021.10.017 PMC8571167

[72] Wang L-L, Zhang C-L. Reply to in vivo confusion over in vivo conversion. Mol Ther. 2022;30(3):986–7. 35108505 10.1016/j.ymthe.2022.01.027 PMC8899670

[73] Geary RS, Norris D, Yu R, Bennett CF. Pharmacokinetics, biodistribution and cell uptake of antisense oligonucleotides. Adv Drug Deliv Rev. 2015;87:46–51. 25666165 10.1016/j.addr.2015.01.008

[74] Egli M, Manoharan M. Chemistry, structure and function of approved oligonucleotide therapeutics. Nucleic Acids Res. 2023;51(6):2529–73. 36881759 10.1093/nar/gkad067 PMC10085713

[75] Guo T, Pan X, Jiang G, Zhang D, Qi J, Shao L, et al. Downregulating PTBP1 fails to convert astrocytes into hippocampal neurons and to alleviate symptoms in Alzheimer’s mouse models. BioRxiv. 2022;28:217. 10.1523/JNEUROSCI.1060-22.2022 PMC9512577 35944999

[76] Guo T, Pan X, Jiang G, Zhang D, Qi J, Shao L, et al. Downregulating PTBP1 fails to convert astrocytes into hippocampal neurons and to alleviate symptoms in alzheimer’s mouse models. J Neurosci. 2022;42(38):7309–17. 35944999 10.1523/JNEUROSCI.1060-22.2022 PMC9512577

[77] Maimon R, Chillon-Marinas C, Vazquez-Sanchez S, Kern C, Jenie K, Malukhina K, et al. Re-activation of neurogenic niches in aging brain. BioRxiv. 2024;133:1710.

[78] Yuan M, Tang Y, Huang T, Ke L, Huang E. In situ direct reprogramming of astrocytes to neurons via polypyrimidine tract-binding protein 1 knockdown in a mouse model of ischemic stroke. Neural Regen Res. 2024;19(10):2240–8. 38488558 10.4103/1673-5374.390957 PMC11034579

[79] Zhu W, Zhou BL, Rong LJ, Ye L, Xu HJ, Zhou Y, et al. Roles of PTBP1 in alternative splicing, glycolysis, and oncogensis. J Zhejiang Univ Sci B. 2020;21(2):122–36. 32115910 10.1631/jzus.B1900422 PMC7076342

[80] Wang G, Moffitt JR, Zhuang X. Multiplexed imaging of high-density libraries of RNAs with MERFISH and expansion microscopy. Sci Rep. 2018;8(1):4847. 29555914 10.1038/s41598-018-22297-7 PMC5859009

[81] Ballas N, Grunseich C, Lu DD, Speh JC, Mandel G. REST and its corepressors mediate plasticity of neuronal gene chromatin throughout neurogenesis. Cell. 2005;121(4):645–57. 15907476 10.1016/j.cell.2005.03.013

[82] Lam XJ, Maniam S, Cheah PS, Ling KH. REST in the road map of brain development. Cell Mol Neurobiol. 2023;43(7):3417–33. 37517069 10.1007/s10571-023-01394-w PMC11410019

[83] Alvarez-Buylla A, Herrera DG, Wichterle H. The subventricular zone: source of neuronal precursors for brain repair. Prog Brain Res. 2000;127:1–11. 11142024 10.1016/s0079-6123(00)27002-7

[84] Doetsch F, Caillé I, Lim DA, García-Verdugo JM, Alvarez-Buylla A. Subventricular zone astrocytes are neural stem cells in the adult mammalian brain. Cell. 1999;97(6):703–16. 10380923 10.1016/s0092-8674(00)80783-7

[85] Young CC, Brooks KJ, Buchan AM, Szele FG. Cellular and molecular determinants of stroke-induced changes in subventricular zone cell migration. Antioxid Redox Signal. 2011;14(10):1877–88. 20673127 10.1089/ars.2010.3435 PMC3078507

[86] David-Bercholz J, Kuo CT, Deneen B. Astrocyte and oligodendrocyte responses from the subventricular zone after injury. Front Cell Neurosci. 2021;15:797553. 35002630 10.3389/fncel.2021.797553 PMC8740317

[87] Chang EH, Adorjan I, Mundim MV, Sun B, Dizon ML, Szele FG. Traumatic brain injury activation of the adult subventricular zone neurogenic niche. Front Neurosci. 2016;10:332. 27531972 10.3389/fnins.2016.00332 PMC4969304

[88] Vandestadt C, Vanwalleghem GC, Khabooshan MA, Douek AM, Castillo HA, Li M, et al. RNA-induced inflammation and migration of precursor neurons initiates neuronal circuit regeneration in zebrafish. Dev Cell. 2021;56(16):2364e8-80e8. 34428400 10.1016/j.devcel.2021.07.021

[89] Wu N, Sun X, Zhou C, Yan J, Cheng C. Neuroblasts migration under control of reactive astrocyte-derived BDNF: a promising therapy in late neurogenesis after traumatic brain injury. Stem Cell Res Ther. 2023;14(1):2. 36600294 10.1186/s13287-022-03232-0 PMC9814466

[90] Kaneko N, Sawada M, Sawamoto K. Mechanisms of neuronal migration in the adult brain. J Neurochem. 2017;141(6):835–47. 28251650 10.1111/jnc.14002

[91] Götz M. Radial glial cells. In: Kettenmann H, Ransom BR, editors. Neuroglia. New York: Oxford University Press; 2012. p. 50–61. 10.1093/med/9780199794591.003.0005.

[92] Miranda-Negrón Y, García-Arrarás JE. Radial glia and radial glia-like cells: Their role in neurogenesis and regeneration. Front Neurosci. 2022;16:1006037. 36466166 10.3389/fnins.2022.1006037 PMC9708897

[93] Zamboni M, Llorens-Bobadilla E, Magnusson JP, Frisén J. A widespread neurogenic potential of neocortical astrocytes is induced by injury. Cell Stem Cell. 2020;27(4):605e5-17e5. 32758425 10.1016/j.stem.2020.07.006 PMC7534841

[94] Nato G, Caramello A, Trova S, Avataneo V, Rolando C, Taylor V, et al. Striatal astrocytes produce neuroblasts in an excitotoxic model of Huntington’s disease. Development. 2015;142(5):840–5. 25655705 10.1242/dev.116657

[95] Schober AL, Wicki-Stordeur LE, Murai KK, Swayne LA. Foundations and implications of astrocyte heterogeneity during brain development and disease. Trends Neurosci. 2022;45(9):692–703. 35879116 10.1016/j.tins.2022.06.009

[96] Yu G, Zhang Y, Ning B. Reactive astrocytes in central nervous system injury: subgroup and potential therapy. Front Cell Neurosci. 2021;15:792764. 35002629 10.3389/fncel.2021.792764 PMC8733560

[97] Miller SJ. Astrocyte heterogeneity in the adult central nervous system. Front Cell Neurosci. 2018;12:401. 30524236 10.3389/fncel.2018.00401 PMC6262303

[98] Liddelow SA, Barres BA. Reactive astrocytes: production, function, and therapeutic potential. Immunity. 2017;46(6):957–67. 28636962 10.1016/j.immuni.2017.06.006

[99] Batiuk MY, Martirosyan A, Wahis J, de Vin F, Marneffe C, Kusserow C, et al. Identification of region-specific astrocyte subtypes at single cell resolution. Nat Commun. 2020;11(1):1220. 32139688 10.1038/s41467-019-14198-8 PMC7058027

[100] Barres BA. The mystery and magic of glia: a perspective on their roles in health and disease. Neuron. 2008;60(3):430–40. 18995817 10.1016/j.neuron.2008.10.013

[101] Herrero-Navarro Á, Puche-Aroca L, Moreno-Juan V, Sempere-Ferràndez A, Espinosa A, Susín R, et al. Astrocytes and neurons share region-specific transcriptional signatures that confer regional identity to neuronal reprogramming. Sci Adv. 2021;7(15):eabe8978. 33827819 10.1126/sciadv.abe8978 PMC8026135

[102] Le Roux PD, Reh TA. Astroglia demonstrate regional differences in their ability to maintain primary dendritic outgrowth from mouse cortical neurons in vitro. J Neurobiol. 1995;27(1):97–112. 7643079 10.1002/neu.480270110

[103] Denis-Donini S, Estenoz M. Interneurons versus efferent neurons: heterogeneity in their neurite outgrowth response to glia from several brain regions. Dev Biol. 1988;130(1):237–49. 3181629 10.1016/0012-1606(88)90430-7

[104] Lanjakornsiripan D, Pior B-J, Kawaguchi D, Furutachi S, Tahara T, Katsuyama Y, et al. Layer-specific morphological and molecular differences in neocortical astrocytes and their dependence on neuronal layers. Nat Commun. 2018;9(1):1623. 29691400 10.1038/s41467-018-03940-3 PMC5915416

[105] Farmer WT, Abrahamsson T, Chierzi S, Lui C, Zaelzer C, Jones EV, et al. Neurons diversify astrocytes in the adult brain through sonic hedgehog signaling. Science. 2016;351(6275):849–54. 26912893 10.1126/science.aab3103

[106] Crooke ST, Baker BF, Crooke RM, Liang XH. Antisense technology: an overview and prospectus. Nat Rev Drug Discov. 2021;20(6):427–53. 33762737 10.1038/s41573-021-00162-z

[107] Li D, Mastaglia FL, Fletcher S, Wilton SD. Progress in the molecular pathogenesis and nucleic acid therapeutics for Parkinson’s disease in the precision medicine era. Med Res Rev. 2020;40(6):2650–81. 32767426 10.1002/med.21718 PMC7589267

[108] Crooke ST, Liang X-h, Crooke RM, Baker BF, Geary RS. Antisense drug discovery and development technology considered in a pharmacological context. Biochem Pharmacol. 2021;189:114196. 32800852 10.1016/j.bcp.2020.114196

[109] Li D, McIntosh CS, Mastaglia FL, Wilton SD, Aung-Htut MT. Neurodegenerative diseases: a hotbed for splicing defects and the potential therapies. Transl Neurodegener. 2021;10(1):16. 34016162 10.1186/s40035-021-00240-7 PMC8136212

[110] Crooke ST, Liang XH, Crooke RM, Baker BF, Geary RS. Antisense drug discovery and development technology considered in a pharmacological context. Biochem Pharmacol. 2021;189:114196. 32800852 10.1016/j.bcp.2020.114196

[111] Zhang MM, Bahal R, Rasmussen TP, Manautou JE, Zhong XB. The growth of siRNA-based therapeutics: updated clinical studies. Biochem Pharmacol. 2021;189:114432. 33513339 10.1016/j.bcp.2021.114432 PMC8187268

[112] Brown KM, Nair JK, Janas MM, Anglero-Rodriguez YI, Dang LTH, Peng H, et al. Expanding RNAi therapeutics to extrahepatic tissues with lipophilic conjugates. Nat Biotechnol. 2022;40(10):1500–8. 35654979 10.1038/s41587-022-01334-x

[113] Hu B, Zhong L, Weng Y, Peng L, Huang Y, Zhao Y, et al. Therapeutic siRNA: state of the art. Signal Transduct Target Ther. 2020;5(1):101. 32561705 10.1038/s41392-020-0207-x PMC7305320

[114] Amiri A, Barreto G, Sathyapalan T, Sahebkar A. siRNA therapeutics: future promise for neurodegenerative diseases. Curr Neuropharmacol. 2021;19(11):1896–911. 33797386 10.2174/1570159X19666210402104054 PMC9185778

[115] Duan C, Kang M, Pan X, Gan Z, Huang V, Li G, et al. Intrathecal administration of a novel siRNA modality extends survival and improves motor function in the SOD1G93A ALS mouse model. Mol Ther Nucleic Acids. 2024;35(1):102147. 38435120 10.1016/j.omtn.2024.102147 PMC10907209

[116] Jinek M, Chylinski K, Fonfara I, Hauer M, Doudna JA, Charpentier E. A programmable dual-RNA–guided DNA endonuclease in adaptive bacterial immunity. Science. 2012;337(6096):816–21. 22745249 10.1126/science.1225829 PMC6286148

[117] Cong L, Ran FA, Cox D, Lin S, Barretto R, Habib N, et al. Multiplex genome engineering using CRISPR/Cas systems. Science. 2013;339(6121):819–23. 23287718 10.1126/science.1231143 PMC3795411

[118] Parums DV. Editorial: first regulatory approvals for CRISPR-Cas9 therapeutic gene editing for sickle cell disease and transfusion-dependent β-thalassemia. Med Sci Monit. 2024;30:e944204. 38425279 10.12659/MSM.944204 PMC10913280

[119] Sheridan C. The world’s first CRISPR therapy is approved: who will receive it? Nat Biotechnol. 2024;42(1):3–4. 37989785 10.1038/d41587-023-00016-6

[120] Pandey S, Gao XD, Krasnow NA, McElroy A, Tao YA, Duby JE, et al. Efficient site-specific integration of large genes in mammalian cells via continuously evolved recombinases and prime editing. Nat Biomed Eng. 2024. 10.1038/s41551-024-01227-1. 38858586 10.1038/s41551-024-01227-1 PMC11754103

[121] Naddaf M. First trial of’base editing’in humans lowers cholesterol-but raises safety concerns. Nature. 2023;623(7988):671–2. 37957349 10.1038/d41586-023-03543-z

[122] Chiesa R, Georgiadis C, Syed F, Zhan H, Etuk A, Gkazi SA, et al. Base-edited CAR7 T cells for relapsed T-Cell acute lymphoblastic leukemia. N Engl J Med. 2023;389(10):899–910. 37314354 10.1056/NEJMoa2300709

[123] Imran Sajid M, Sultan Sheikh F, Anis F, Nasim N, Sumbria RK, Nauli SM, et al. siRNA drug delivery across the blood–brain barrier in Alzheimer’s disease. Adv Drug Deliv Rev. 2023;199:114968. 37353152 10.1016/j.addr.2023.114968 PMC10528676

[124] Min HS, Kim HJ, Naito M, Ogura S, Toh K, Hayashi K, et al. Systemic brain delivery of antisense oligonucleotides across the blood-brain barrier with a glucose-coated polymeric nanocarrier. Angew Chem Int Ed Engl. 2020;59(21):8173–80. 31995252 10.1002/anie.201914751 PMC7317551

[125] Godfrey C, Desviat LR, Smedsrød B, Piétri-Rouxel F, Denti MA, Disterer P, et al. Delivery is key: lessons learnt from developing splice-switching antisense therapies. EMBO Mol Med. 2017;9(5):545–57. 28289078 10.15252/emmm.201607199 PMC5412803

[126] Yeoh YQ, Amin A, Cuic B, Tomas D, Turner BJ, Shabanpoor F. Efficient systemic CNS delivery of a therapeutic antisense oligonucleotide with a blood-brain barrier-penetrating ApoE-derived peptide. Biomed Pharmacother. 2024;175:116737. 38749176 10.1016/j.biopha.2024.116737

[127] Goyenvalle A, Griffith G, Babbs A, Andaloussi SE, Ezzat K, Avril A, et al. Functional correction in mouse models of muscular dystrophy using exon-skipping tricyclo-DNA oligomers. Nat Med. 2015;21(3):270–5. 25642938 10.1038/nm.3765

[128] Relizani K, Griffith G, Echevarría L, Zarrouki F, Facchinetti P, Vaillend C, et al. Efficacy and safety profile of tricyclo-DNA antisense oligonucleotides in Duchenne muscular dystrophy mouse model. Mol The Nucleic Acids. 2017;8:144–57. 28918017 10.1016/j.omtn.2017.06.013 PMC5498286

[129] Hariharan VN, Shin M, Chang CW, O’Reilly D, Biscans A, Yamada K, et al. Divalent siRNAs are bioavailable in the lung and efficiently block SARS-CoV-2 infection. Proc Natl Acad Sci USA. 2023;120(11):e2219523120. 36893269 10.1073/pnas.2219523120 PMC10089225

[130] Cheng S-Y, Caiazzi J, Biscans A, Alterman JF, Echeverria D, McHugh N, et al. Single intravitreal administration of a tetravalent siRNA exhibits robust and efficient gene silencing in mouse and pig photoreceptors. Mol Ther Nucleic Acids. 2024;35(1):102. 10.1016/j.omtn.2023.102088 PMC10772295 38192611

[131] Alterman JF, Godinho BMDC, Hassler MR, Ferguson CM, Echeverria D, Sapp E, et al. A divalent siRNA chemical scaffold for potent and sustained modulation of gene expression throughout the central nervous system. Nat Biotechnol. 2019;37(8):884–94. 31375812 10.1038/s41587-019-0205-0 PMC6879195

[132] Xiao H, Amarsaikhan O, Zhao Y, Yu X, Hu X, Han S, et al. Astrocyte-targeted siRNA delivery by adenosine-functionalized LNP in mouse TBI model. Mol Ther Nucleic Acids. 2023;34:12. 10.1016/j.omtn.2023.102065 PMC10661454 38028196

[133] Zhu J, Qiu W, Wei F, Wang Y, Wang Q, Ma W, et al. Reactive A1 astrocyte-targeted nucleic acid nanoantiepileptic drug downregulating adenosine kinase to rescue endogenous antiepileptic pathway. ACS Appl Mater Interfaces. 2023;15(25):29876–88. 37334941 10.1021/acsami.3c03455

[134] Soltani Dehnavi S, Eivazi Zadeh Z, Harvey AR, Voelcker NH, Parish CL, Williams RJ, et al. Changing fate: reprogramming cells via engineered nanoscale delivery materials. Adv Mater. 2022;34(33):2108757. 10.1002/adma.202108757 35396884

[135] Mahmoudi N, Wang Y, Moriarty N, Ahmed NY, Dehorter N, Lisowski L, et al. Neuronal replenishment via hydrogel-rationed delivery of reprogramming factors. ACS Nano. 2024;18(4):3597–613. 38221746 10.1021/acsnano.3c11337

[136] Foust KD, Nurre E, Montgomery CL, Hernandez A, Chan CM, Kaspar BK. Intravascular AAV9 preferentially targets neonatal neurons and adult astrocytes. Nat Biotechnol. 2009;27(1):59–65. 19098898 10.1038/nbt.1515 PMC2895694

[137] Belur LR, Podetz-Pedersen KM, Tran TA, Mesick JA, Singh NM, Riedl M, et al. Intravenous delivery for treatment of mucopolysaccharidosis type I: a comparison of AAV serotypes 9 and rh10. Mol Genet Metab Rep. 2020;24:100604. 32461912 10.1016/j.ymgmr.2020.100604 PMC7242863

[138] Davidson BL, Stein CS, Heth JA, Martins I, Kotin RM, Derksen TA, et al. Recombinant adeno-associated virus type 2, 4, and 5 vectors: transduction of variant cell types and regions in the mammalian central nervous system. Proc Natl Acad Sci U S A. 2000;97(7):3428–32. 10688913 10.1073/pnas.050581197 PMC16256

[139] Meneghini V, Peviani M, Luciani M, Zambonini G, Gritti A. Delivery platforms for CRISPR/Cas9 genome editing of glial cells in the central nervous system. Front Genome Ed. 2021;3:644319. 34713256 10.3389/fgeed.2021.644319 PMC8525379

[140] Song R, Pekrun K, Khan TA, Zhang F, Paşca SP, Kay MA. Selection of rAAV vectors that cross the human blood-brain barrier and target the central nervous system using a transwell model. Mol Ther Methods Clin Dev. 2022;27:73–88. 36186955 10.1016/j.omtm.2022.09.002 PMC9494039

